# Infection Control Performance Related to Emerging Infectious Diseases Among Emergency Department Nurses: An Ecological Systems Theory‐Based Cross‐Sectional Study

**DOI:** 10.1155/jonm/6160239

**Published:** 2026-07-29

**Authors:** Yu Jin Park, Hye Young Kim

**Affiliations:** ^1^ College of Nursing, Jeonbuk National University, Baekje-daero, Deokjin-gu, Jeonju-si, Jeonbuk-do, Republic of Korea, cbnu.edu; ^2^ College of Nursing, Research Institute of Nursing Science, Jeonbuk National University, Baekje-daero, Deokjin-gu, Jeonju-si, Jeonbuk-do, Republic of Korea, cbnu.edu

**Keywords:** communicable diseases, emerging, infection control, nurses, safety management

## Abstract

**Background:**

Emergency department (ED) nurses face elevated risks of infection during emerging infectious disease (EID) outbreaks. A multilevel understanding of factors influencing infection control performance is essential for improving safety in these settings.

**Aim(s):**

To examine associations among safety control, communication, perceived patient safety culture, and infection control performance among ED nurses and identify key influencing factors based on ecological systems theory.

**Methods:**

A cross‐sectional descriptive study was conducted. Data were collected in October 2023 from 227 ED nurses at tertiary hospitals in South Korea who had experience caring for patients with confirmed or suspected EIDs. Structured, self‐administered online questionnaires were used, and the responses were analyzed using descriptive statistics, *t*‐tests, one‐way ANOVA, correlation analysis, and hierarchical regression (SPSS Version 28).

**Results:**

Communication exhibited the strongest association with infection control performance (*β* = 0.53, *p* < 0.001), followed by safety control (*β* = 0.27, *p* < 0.001) and the availability of negative‐pressure isolation rooms with anterooms (3–4) (*β* = 0.13, *p* = 0.016). These factors explained 74.0% of the variance in infection control performance (*F* = 46.83, *p* < 0.001).

**Conclusion:**

Infection control performance among ED nurses was associated with individual, organizational, and environmental factors. Ecological systems theory provided an effective framework for identifying these multilevel influences.

**Implications for Nursing Management:**

Nurse managers should implement comprehensive multilevel strategies addressing safety control at the microsystem, communication at the mesosystem, and infrastructure at the macrosystem to improve infection control performance. This study provides evidence to support targeted interventions in emergency settings.

## 1. Introduction

Emerging infectious diseases (EIDs) are defined as infections that appear in a population for the first time or have previously existed but are rapidly increasing in incidence or geographic spread [1]. Examples include severe acute respiratory syndrome (SARS), Middle East Respiratory Syndrome (MERS), Ebola virus disease, and coronavirus disease 2019 (COVID‐19). The recent surge in both new and resurgent infectious diseases presents a significant threat to public health and socioeconomic stability at national and global levels, highlighting the critical importance of effective infectious disease prevention and management strategies [2].

Emergency department (ED) nurses frequently come into direct contact with patients, placing them at increased risk of acquiring infections or transmitting pathogens to others [3–5]. During outbreaks of EIDs, maintaining high standards of infection control among ED healthcare workers is particularly challenging due to high patient density, prolonged patient boarding, and frequent disruptions in medical care delivery [6–8]. Thus, continuous improvement in infection control performance among ED nurses is essential. Identifying multiple influencing factors at various ecological levels can enhance infection control practices in emergency settings.

Recent studies have suggested that infection prevention and control (IPC) performance among nurses is influenced not only by individual knowledge and attitudes but also by organizational and environmental contexts. Nurses in hospital settings have reported barriers related to workplace conditions and institutional support that may affect adherence to IPC practices [9–11]. These findings support the need to examine infection control performance from a multilevel perspective.

**FIGURE 1:**
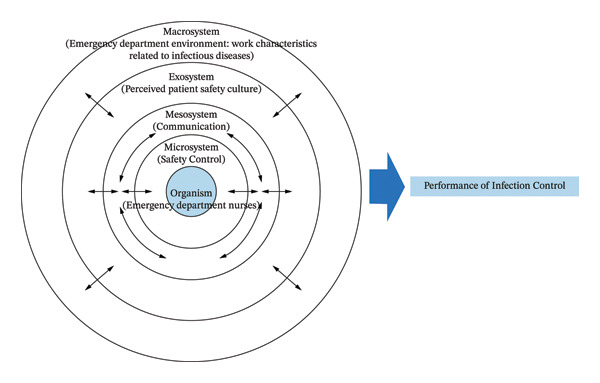
Theoretical framework of this study.

To comprehensively understand these multifaceted influences, this study adopts Bronfenbrenner’s ecological systems theory [12], which conceptualizes human behavior as shaped by ongoing interactions between individuals and their surrounding environments. This framework identifies four interrelated systems—the microsystem, mesosystem, exosystem, and macrosystem—with the individual at the center as the organism (Figure 1). In addition, the theory also encompasses a dimension of time, referred to as the chronosystem, which reflects changes and transitions over time. In this study, ED nurses are regarded as the organism, and their infection control performance is examined in relation to these ecological systems. Applying this model allows for an integrated understanding of how personal, organizational, and societal factors jointly influence ED nurses’ behaviors in the context of EIDs.

Safety control, defined as nurses’ cognitive ability to influence safe outcomes in their work environment, is categorized within the microsystem [13]. It has been positively associated with patient safety behaviors and functions as a mediator between organizational safety climate and actual safety [14, 15]. Higher levels of safety control have been shown to improve performance in patient safety‐related nursing activities [16].

Communication within healthcare institutions, referring to the timely and accurate exchange of information between healthcare organizations and their staff, is situated within the mesosystem [17]. Particularly during outbreaks of EIDs, when infection control guidelines change frequently, communication failures have been identified as a key factor contributing to discrepancies between prescribed protocols and actual nursing practices in emergency settings [5, 18, 19].

Perceived patient safety culture, conceptualized as the shared organizational values, beliefs, norms, and behaviors that shape safety‐related expectations and practices [20, 21], belongs to the exosystem because it reflects broader organizational environments that indirectly influence nurses’ infection control performance beyond direct interpersonal interactions. In emergency settings, where unpredictable conditions and high demands are common, fostering a strong perceived patient safety culture among ED nurses has been shown to promote patient safety activities and improve infection control performance, underscoring its critical role in managing EIDs [22].

Work characteristics related to EIDs, including institutional preparedness, personal protective equipment (PPE) supply, infection control education, and structured monitoring, belong to the macrosystem. These can significantly influence ED nurses’ infection control performance. Effective ED management for EIDs includes environmental control measures and staff education specific to emerging infections [23]. Infection control performance improves when nurses receive structured monitoring and feedback on PPE use [17]. Overcrowding in ED settings, characterized by prolonged sharing of spaces among healthcare providers, patients, and caregivers, increases the risk of hospital‐acquired infections through environmental transmission [24, 25].

This study aims to comprehensively examine factors influencing ED nurses’ infection control performance regarding EIDs through an ecological systems approach. Specifically, it seeks to analyze relationships among variables across the microsystem, mesosystem, exosystem, and macrosystem, identifying key influences at each level (Figure 1).

The findings of this study will provide foundational data for improving ED nurses’ working environments and contribute to the development of safer emergency medical systems. Additionally, the results will guide future research, inform nursing practice and education, and support evidence‐based policy decisions, including the efficient allocation of healthcare resources and personnel, as well as system restructuring efforts to effectively manage EIDs.

## 2. Materials and Methods

### 2.1. Aims and Hypotheses

This study aimed to investigate ED nurses’ safety control, communication, perceived patient safety culture, and infection control performance, as well as to identify associated factors based on ecological systems theory.

The study hypotheses were as follows: (H1) There is a relationship between safety control (microsystem) and infection control performance; (H2) there is a relationship between communication (mesosystem) and infection control performance; (H3) there is a relationship between perceived patient safety culture (exosystem) and infection control performance; and (H4) there is a relationship between work characteristics related to EIDs (macrosystem) and infection control performance.

### 2.2. Study Design

A descriptive, cross‐sectional design was adopted. To ensure methodological rigor and transparency, the study adhered to the STROBE (Strengthening the Reporting of Observational Studies in Epidemiology) guidelines.

### 2.3. Participants

Convenience sampling was employed to recruit ED nurses working at tertiary hospitals in South Korea. The inclusion criteria were as follows: (1) ED nurses who had been working at tertiary hospitals in South Korea for more than 1 year after obtaining their nursing license; (2) nurses who had directly cared for patients with confirmed or suspected EIDs within the past year; and (3) nurses who provided written informed consent to participate in the study. The required sample size was calculated using G∗Power 3.1.9.7 software. For a hierarchical regression analysis with 24 predictors, assuming a medium effect size (*f* ^2^ = 0.15), a significance level of 0.05, and a power of 0.90, a minimum of 206 participants was required [26]. Considering a potential dropout rate of approximately 15%, 243 responses were collected through the online survey. After excluding 16 participants who did not meet the inclusion criteria, data from 227 participants were included in the final analysis.

### 2.4. Measurements

Data were collected using a structured questionnaire composed of five sections. The instruments used to measure infection control performance, perceived safety control, communication, and perceived patient safety culture were employed with permission from the original developers.

#### 2.4.1. Infection Control Performance

Infection control performance was measured using a tool originally developed to assess adherence to MERS isolation guidelines [27], which was later modified to align with COVID‐19 response guidelines [28] and validated by experts [17]. The revised instrument was adapted for use with ED nurses and modified to use a Likert scale based on expert review. The instrument was originally developed in Korean; therefore, no translation or back‐translation process was required. The final version was validated by a panel of seven experts, including three nursing professors, one infectious disease physician, one infection control manager, and two ED nurses. As a result of this process, the item‐level content validity index (CVI) was ≥ 0.78 for all items, and the scale‐level CVI was ≥ 0.80, indicating good content validity [29]. The final tool consists of 18 items rated on a 5‐point Likert scale. Fourteen items assess direct nursing care (e.g., isolation, PPE, specimen handling) and four items assess indirect nursing care (e.g., visitor control, waste management) using a 5‐point Likert scale. Subscale scores were calculated as the sum of item responses, with possible score ranges of 14–70 for direct nursing care and 4–20 for indirect nursing care. Higher scores indicate better infection control performance. The internal consistency in this study was high (Cronbach’s *α* = 0.94). No reverse‐coded items were included.

#### 2.4.2. Safety Control

Safety control was measured using a 7‐item instrument originally developed by Anderson et al. [13] and adapted for healthcare professionals by Jeong [30]. The Korean version adapted by Jeong [30] was used in this study. Therefore, no additional translation or back‐translation process was required. This 7‐item instrument assesses the extent to which individuals feel capable of maintaining safety. Responses are rated on a 5‐point Likert scale, with higher scores indicating stronger safety control (Cronbach’s *α* = 0.84 in this study). No reverse‐coded items were included.

#### 2.4.3. Communication

Communication within healthcare institutions for EID management was measured using a 9‐item scale developed by Yi and Cha [17], based on the Crisis and Emergency Risk Communication guidelines from the Centers for Disease Control and Prevention (CDC) [31] and the KDCA’s standard operating procedures [32]. The tool was originally developed in Korean. Therefore, no translation or back‐translation process was required. The tool assesses nurses’ perceptions of communication processes across departments and units within the hospital, reflecting interunit interactions relevant to EID management, which aligns with the mesosystem level of Bronfenbrenner’s ecological framework. The tool comprises six items on communication standards (timeliness, reliability, clear action guidelines, and continuity) and three items on communication infrastructure (practical focus, interdepartmental agreement). Each item is rated on a 5‐point Likert scale; higher scores reflect more effective institutional communication (Cronbach’s *α* = 0.89 in this study). No reverse‐coded items were included.

#### 2.4.4. Perceived Patient Safety Culture

Perceived patient safety culture was assessed using the Korean version of the Hospital Survey on Patient Safety Culture, originally developed by the Agency for Healthcare Research and Quality (AHRQ) [23] and translated/adapted by Kim et al. [33] for nurses in tertiary hospitals. The Korean version by Kim et al. [33] was used in this study. Therefore, no additional translation or back‐translation process was required. The tool consists of 44 items across six subscales. Each item is rated on a 5‐point Likert scale. Negatively worded items were reverse‐coded prior to analysis. Higher scores represent more positive perceptions of patient safety culture (Cronbach’s *α* = 0.90 in this study).

#### 2.4.5. Demographic and EID‐Related Work Characteristics

This section included five demographic items (e.g., gender, age, education, marital status, and current position) and 14 items related to EID‐related work characteristics. These included ED work experience, institutional resources (e.g., deployment of dedicated staff, availability of appropriate PPE, number of negative‐pressure rooms including anterooms), and personal experiences with EIDs (e.g., care for confirmed or suspected patients before COVID‐19, personal infection experience, quarantine experience, number of times received EID‐related education, PPE education, PPE retraining during the EIDs pandemic, and monitoring/feedback on PPE donning/doffing from infection control staff). Average daily working hours before and during the pandemic (excluding rest time) were also included. Some variables (e.g., daily working hours) were dichotomized (≤ 8 vs. > 8 h) based on standard clinical work shift durations in nursing practice.

### 2.5. Data Collection

Data for this study were collected through an online survey conducted from October 17 to October 31, 2023. Prior to data collection, permission was obtained to post recruitment notices on online communities and social networking services commonly used by nurses. The recruitment notice included information about the study’s topic and purpose, inclusion criteria, participation procedures, and the estimated time required to complete the survey. Nurses who were interested in participating could access the survey via a provided online link or QR code. The first page of the online survey served as a screening tool to verify the inclusion criteria. A total of 243 individuals accessed and completed the survey. However, 16 were excluded after the screening process because they did not meet the specific criteria (less than one year of clinical experience). Consequently, a total of 227 responses were collected, all of which were completed without any missing data and included in the final analysis.

### 2.6. Ethical Considerations

This study was approved by the Institutional Review Board of Jeonbuk National University (IRB) prior to data collection (IRB No. JBNU 2023‐08‐012‐005). The first page of the online survey explained the study’s purpose, procedures, and data confidentiality. Participants provided informed consent by checking a box labeled “I agree to participate in the study.” All responses were used solely for research purposes, and data were encrypted and securely stored, accessible only to the research team. The data will be retained for 3 years in accordance with IRB regulations.

### 2.7. Data Analysis

Data were analyzed using SPSS Version 28.0. All tests were two‐sided, and statistical significance was set at *p* < 0.05. Participants’ demographic and work‐related characteristics associated with infectious diseases were analyzed using descriptive statistics. Levels of safety control, communication, perceived patient safety culture, and infection control performance were summarized using means, standard deviations, ranges, and minimum and maximum values. Normality was assessed using skewness and kurtosis. Variations in infection control performance based on sociodemographic characteristics and infectious disease‐related work factors were analyzed using independent *t*‐tests, one‐way ANOVAs, and Welch tests, followed by Scheffé and Games‐Howell post hoc analyses as appropriate. Pearson’s correlation coefficients were used to examine relationships among safety control, communication, perceived patient safety culture, and infection control performance. Hierarchical multiple regression analysis was performed to assess the associations of the variables outlined in the conceptual framework—safety control, communication, and perceived patient safety culture—with infection control performance.

## 3. Results

### 3.1. Participants’ Sociodemographic Characteristics and Work Characteristics Related to Infectious Diseases

The mean age of participants was 30.56 years (range: 25–58), and the majority were women (*n* = 214, 94.3%). A total of 188 participants (82.8%) held a bachelor’s degree, 186 (81.9%) were unmarried, and most participants were staff nurses (*n* = 194, 85.5%) (Table 1).

**TABLE 1:** Infection control performance according to sociodemographic characteristics of the participants (*N* = 227).

Characteristics	Categories	n (%)	Infection control performance		
			Mean ± SD	t or F	*p*
Gender	Women	214 (94.3)	69.90 ± 11.99	0.33	0.741
	Men	13 (5.7)	68.77 ± 12.89		

Age (year)	25–29	106 (46.7)	68.83 ± 13.78	1.04^†^	0.386
	30–34	116 (51.1)	70.60 ± 10.30		
	≥ 35	5 (2.2)	73.60 ± 7.73		

Education level	Three‐year diploma	24 (10.6)	67.71 ± 12.87	0.83	0.436
	Bachelor’s degree	188 (82.8)	69.88 ± 12.02		
	≥ Master’s degree	15 (6.6)	72.80 ± 10.58		

Marital status	Unmarried	186 (81.9)	70.21 ± 12.28	0.98	0.326
	Married	41 (18.1)	68.17 ± 10.72		

Position	Staff nurse	194 (85.5)	69.88 ± 11.69	0.09^†^	0.926
	Charge nurse	33 (14.5)	69.64 ± 14.03		

*Abbreviation:* SD, standard deviation.

^†^Welch test.

Regarding work characteristics related to infectious diseases, most participants had less than 5 years of ED experience (*n* = 164, 72.2%), reported having dedicated personnel for infection control (*n* = 188, 82.8%), and had access to a sufficient supply of PPE (*n* = 199, 87.7%). The most common number of negative‐pressure isolation rooms was five or more (*n* = 112, 49.3%), and 102 participants (44.9%) had five or more such rooms, including anterooms, followed by 91 (40.1%) with 3–4, and 34 (15.0%) with 1–2; none reported having zero rooms. Regarding previous experience with EIDs, 154 (67.8%) had cared for EID patients prior to COVID‐19. A total of 170 (74.9%) had been infected with an EID, 189 (83.3%) had experienced self‐isolation, 33 (14.5%) had received fewer than two EID‐related education sessions, and 40 (17.6%) had received fewer than two PPE donning and doffing education sessions. PPE retraining during an EID outbreak was reported by 170 participants (74.9%), and 189 (83.3%) received monitoring and feedback for donning and doffing PPE. Before the EID outbreak, 122 participants (53.7%) worked less than 8 h per day (excluding breaks), whereas during the outbreak, 190 (83.7%) worked more than 8 h (Table 2).

**TABLE 2:** Infection control performance according to work characteristics related to infectious diseases of the participants (*N* = 227).

Characteristics	Categories	*n* (%)	Infection control performance		
			Mean ± SD	t or F	*p* (Scheffé or Games‐Howell)
Experience in ED (years)	< 5	164 (72.2)	69.16 ± 11.98	−1.38	0.168
	≥ 5	63 (27.8)	71.62 ± 12.03		

Existence of personnel dedicated	Yes	188 (82.8)	71.99 ± 11.07	6.44	< 0.001
	No	39 (17.2)	59.46 ± 11.04		

Sufficient supply of suitable PPE	Yes	199 (87.7)	70.95 ± 11.62	3.82	< 0.001
	No	28 (12.3)	61.96 ± 12.06		

Number of negative‐pressure isolation room	1–2	17 (7.5)	68.82 ± 10.75	0.49^†^	0.616
	3–4	98 (43.2)	70.70 ± 10.65		
	≥ 5	112 (49.3)	69.24 ± 13.30		

Number of negative‐pressure isolation room (with anteroom)	1–2^a^	34 (15.0)	69.00 ± 12.39	10.15^†^	< 0.001
	3–4^b^	91 (40.1)	66.07 ± 12.40		(b < c)
	≥ 5^c^	102 (44.9)	73.49 ± 10.46		

Experience of EID patients care prior to COVID‐19	Yes	154 (67.8)	72.21 ± 10.22	4.04^†^	< 0.001
	No	73 (32.2)	64.84 ± 13.92		

Experience of being infected with EID	Yes	170 (74.9)	68.69 ± 11.99	−2.53	0.012
	No	57 (25.1)	73.28 ± 11.53		

Experience of self‐isolation	Yes	189 (83.3)	69.70 ± 11.53	−0.33	0.741
	No	38 (16.7)	70.53 ± 14.36		

Number of EID education	< 2	33 (14.5)	67.55 ± 10.14	−1.36^†^	0.180
	≥ 2	194 (85.5)	70.23 ± 12.29		

Number of education for donning and doffing of PPE	< 2	40 (17.6)	66.35 ± 9.30	−2.45^†^	0.017
	≥ 2	187 (82.4)	70.59 ± 12.41		

PPE retraining during EID outbreak	Yes	170 (74.9)	71.32 ± 11.68	3.27	0.001
	No	57 (25.1)	65.44 ± 12.02		

Monitoring and feedback for donning and doffing of PPE	Yes	189 (83.3)	71.97 ± 11.10	6.46	< 0.001
	No	38 (16.7)	59.26 ± 10.85		

Working hours prior to EID outbreak (hour/day)	≤ 8	122 (53.7)	74.79 ± 9.78	7.34^†^	< 0.001
	> 8	105 (46.3)	64.10 ± 11.84		

Working hours during EID outbreak (hour/day)	≤ 8	37 (16.3)	76.00 ± 10.77	3.49	0.001
	> 8	190 (83.7)	68.64 ± 11.90		

*Abbreviations:* COVID‐19, coronavirus disease 2019; ED, emergency department; EID, emerging infectious disease; PPE, personal protective equipment.

^†^Welch test.

### 3.2. Infection Control Performance According to Sociodemographic Characteristics and Work Characteristics Related to Infectious Diseases

There were no statistically significant differences in infection control performance based on gender, age, education level, marital status, or job position (Table 1).

Infection control performance was significantly higher among participants who had worked in units with dedicated personnel (*t* = 6.44, *p* < 0.001) and who had sufficient PPE supply (*t* = 3.82, *p* < 0.001). Post hoc analysis showed that participants with five or more negative‐pressure isolation rooms, including anterooms, had significantly higher infection control performance than those with 3–4 such rooms (*F* = 10.15, *p* < 0.001). Infection control performance was also significantly higher among those with prior EID care experience (*t* = 4.04, *p* < 0.001), no infection history (*t* = −2.53, *p* = 0.012), more than two PPE education sessions (*t* = −2.45, *p* = 0.017), PPE retraining (*t* = 3.27, *p* = 0.001), PPE monitoring and feedback (*t* = 6.46, *p* < 0.001), working less than 8 h per day before the outbreak (*t* = 7.34, *p* < 0.001), and working less than 8 h per day during the outbreak (*t* = 3.49, *p* = 0.001). There were no significant differences in the total number of negative‐pressure rooms, self‐isolation experience, or the number of EID‐related education sessions (Table 2).

### 3.3. Scores of Safety Control, Communication, Perceived Patient Safety Culture, and Infection Control Performance to EID

The mean safety control score was 25.96 ± 4.68 (range: 7–35). The mean communication score was 34.38 ± 6.32 (range: 9–45), and the mean perceived patient safety culture score was 144.11 ± 19.26 (range: 44–220). The mean infection control performance score was 69.84 ± 12.01 (range: 18–90), with a mean score of 54.22 ± 9.33 (range: 33–70) for direct nursing care and 15.62 ± 2.99 (range: 8–20) for indirect nursing care. All variables used in the study demonstrated normal distribution, with absolute values of skewness and kurtosis less than ± 2 (Table 3).

**TABLE 3:** Descriptive statistics among study variables (*N* = 227).

Variables	Mean ± SD	Min	Max	Possible range	Skewness	Kurtosis
Safety control	25.96 ± 4.68	11	35	7–35	−0.38	−0.32
Communication	34.38 ± 6.32	20	45	9–45	−0.49	−0.89
Perceived patient safety culture	144.11 ± 19.26	94	204	44–220	0.47	0.40
Infection control performance	69.84 ± 12.01	42	90	18–90	−0.40	−0.85
Direct nursing care	54.22 ± 9.33	33	70	33–70		
Indirect nursing care	15.62 ± 2.99	8	20	8–20		

*Abbreviation:* SD, standard deviation.

### 3.4. Relationship Between Safety Control, Communication, Perceived Patient Safety Culture, and Infection Control Performance

Partial correlation analysis was conducted to examine the relationships between safety control, communication, perceived patient safety culture, and infection control performance, while controlling for variables that were significantly associated with infection control performance in Table 2: existence of personnel dedicated, sufficient supply of suitable PPE, number of negative‐pressure isolation room (with anterooms), experience of EID patients care prior to COVID‐19, experience of being infected with EID, number of education for donning and doffing of PPE, PPE retraining during EID outbreak, monitoring and feedback for donning and doffing of PPE, working hours prior to EID outbreak (hour/day), and working hours during EID outbreak (hour/day). Infection control performance showed significant positive correlations with safety control (*r* = 0.75, *p* < 0.001, 95% CI [0.69, 0.80]), communication (*r* = 0.83, *p* < 0.001, 95% CI [0.78, 0.87]), and perceived patient safety culture (*r* = 0.68, *p* < 0.001, 95% CI [0.61, 0.75]).

### 3.5. Multiple Regression Results With Infection Control Performance as Dependent Variables

Hierarchical regression analysis was conducted based on Bronfenbrenner’s ecological systems theory. Variables that showed significant differences according to infection control performance were entered as independent variables. The microsystem variable “safety control” was entered first, followed by the mesosystem variable “communication,” the exosystem variable “perceived patient safety culture,” and finally, the macrosystem variables that were significantly associated with infection control performance. The macrosystem variables—including the presence of dedicated personnel, availability of PPE, number of negative‐pressure isolation rooms (with anterooms), prior experience caring for EID patients before COVID‐19, personal history of EID infection, PPE education, PPE retraining, PPE monitoring and feedback, and working hours before and during the EID outbreak—were dummy‐coded for analysis.

To test the assumption of the linear regression, the normality and linearity of all variables were examined, and the Durbin–Watson statistic was 1.77, close to the cutoff of 2, thereby eliminating the issue of autocorrelation. Equal variance and normality of residuals were tested with a standardized residual Q–Q plot, and all variables were independent, with an absolute correlation coefficient ranging from 0.80 or less. Tolerance values were all above 0.10, with a range of 0.31–0.81, and variance inflation factors were all below 10, which ruled out the issue of multicollinearity [34].

Table 4 presents the results of the hierarchical regression analysis. Model 1, which included participants’ safety control, was statistically significant (*F* = 296.79, *p* < 0.001) and explained approximately 56.7% of the adjusted *R* ^2^ in infection control performance. Model 2, communication was added and the model remained significant (*F* = 303.81, *p* < 0.001), with the adjusted *R* ^2^ increasing to 72.8%. In this model, communication (*β* = 0.59, *p* < 0.001) and safety control (*β* = 0.32, *p* < 0.001) were both significant predictors. Model 3 included perceived patient safety culture in addition to the previous variables and was also statistically significant (*F* = 202.64, *p* < 0.001), with the adjusted *R* ^2^ remaining at 72.8%. In this model, communication (*β* = 0.58, *p* < 0.001) and safety control (*β* = 0.29, *p* < 0.001) remained significant, whereas perceived patient safety culture was not a significant predictor (*β* = 0.05, *p* = 0.368). Model 4 incorporated work characteristics related to infectious diseases and was statistically significant (*F* = 46.83, *p* < 0.001), with the adjusted *R* ^2^ increasing to 74.0%. In this model, communication (*β* = 0.53, *p* < 0.001), safety control (*β* = 0.27, *p* < 0.001), and negative‐pressure isolation rooms with anterooms (3–4) (*β* = 0.13, *p* = 0.016) were significant predictors. Perceived patient safety culture (*β* = 0.05, *p* = 0.368) remained nonsignificant.

**TABLE 4:** Multiple regression results with infection control performance as dependent variables (*N* = 227).

Variables	Infection control performance															
	Model 1				Model 2				Model 3				Model 4			
	*B*	*β*	SE	*p*	*B*	*β*	SE	*p*	*B*	*β*	SE.	*p*	*B*	*β*	SE.	*p*
				95% CI				95% CI				95% CI				95% CI
Constant	19.62		2.96	< 0.001	9.90		2.49	< 0.001	8.16		3.15	0.010	9.64		3.46	0.006
				13.79–25.46				4.99–14.81				1.96–14.37				2.83–16.46

Safety control	1.94	0.75	0.11	< 0.001	0.82	0.32	0.13	< 0.001	0.75	0.29	0.15	< 0.001	0.69	0.27	0.16	< 0.001
				1.71–2.16				0.56–1.08				0.45–1.05				0.38–1.00

Communication					1.13	0.59	0.10	< 0.001	1.10	0.58	0.10	< 0.001	1.01	0.53	0.12	< 0.001
								0.94–1.32				0.89–1.30				0.78–1.24

Perceived patient safety culture									0.03	0.05	0.14	0.368	0.01	0.01	0.04	0.880
												−0.04–0.10				−0.07–0.08

Personnel dedicated (yes; ref: no)^+^													1.62	0.05	1.23	0.191
																−0.81–4.04

PPE supply (yes; ref: no)^∗^													0.47	0.01	1.38	0.735
																−2.24–3.18

Negative‐pressure isolation room (with anteroom) (3–4; ref: 1–2)^∗^													3.08	0.13	1.26	0.016
																0.59–5.57

Negative‐pressure isolation room (with anteroom) (≥ 5; ref: 1–2)^∗^													0.60	0.02	1.28	0.643
																−1.93–3.13

EID care pre‐COVID‐19 (yes; ref: no)^∗^													−0.38	−0.02	0.99	0.703
																−2.33–1.58

Infected with EID (no; ref: yes)^∗^													1.15	0.04	1.04	0.270
																−0.90–3.21

PPE education (≥ 2; ref: < 2)^∗^													1.12	0.04	1.19	0.347
																−1.22–3.46

PPE retraining (yes; ref: no)^∗^													0.85	0.03	1.11	0.445
																−1.34–3.05

PPE monitoring and feedback (yes; ref: no)^∗^													1.25	0.04	1.27	0.328
																−1.26–3.76

Working hours prior to EID (≤ 8; ref: > 8)^∗^													1.23	0.05	0.10	0.219
																−0.74–3.20

Working hours during EID (≤ 8; ref: > 8)^∗^													0.35	0.01	1.27	0.782
																−2.15–2.85

*R* ^2^	0.569				0.731				0.732				0.756			

Adjusted *R* ^2^	0.567				0.728				0.728				0.740			

△Adjusted *R* ^2^	0.569				0.162				0.001				0.024			

*F*	296.79				303.81				202.64				46.83			

*p*	< 0.001				< 0.001				< 0.001				< 0.001			

*Abbreviations: B*, unstandardized estimates; SE, standard error; *β*, standardized estimates; CI, confidence interval; COVID‐19, coronavirus disease 2019; EID, emerging infectious disease; PPE, personal protective equipment.

^∗^The references were personnel dedicated (no), PPE supply (no), negative‐pressure isolation room (with anteroom) (1–2), EID care pre‐COVID‐19 (no), infected with EID (yes), PPE education (< 2), PPE retraining (no), PPE monitoring and feedback (no), working hours prior to EID (> 8), and working hours during EID (> 8).

Based on these findings, Hypotheses 1, 2, and 4 were supported, indicating that safety control, communication, and certain work‐related characteristics (i.e., negative‐pressure isolation rooms with anterooms) were significantly associated with infection control performance. In contrast, Hypothesis 3 was not supported, as perceived patient safety culture was not significantly associated with infection control performance.

## 4. Discussion

This study, grounded in Bronfenbrenner’s ecological systems theory (1979), aimed to investigate factors influencing infection control performance among ED nurses during EID situations. Specifically, it assessed infection control performance and identified the roles of safety control, communication, and perceived patient safety culture. The findings revealed that communication and safety control were significantly associated with infection control performance, and that EDs with 3–4 negative‐pressure isolation rooms demonstrated higher performance levels. These results are consistent with prior studies; for instance, Yi and Cha [17] identified communication and PPE monitoring as influential factors, and Park and Seo [35] highlighted the importance of organizational culture. Together, these findings underscore the complex, multidimensional nature of infection control performance, which is associated with individual, organizational, and environmental systems. Therefore, it is crucial to approach intervention development from an ecological perspective that integrates these multiple layers.

To further explore these relationships, hierarchical regression analysis was conducted by sequentially entering variables from the microsystem (safety control), mesosystem (communication), exosystem (perceived patient safety culture), and macrosystem (work characteristics). This model explained 74.0% of the variance in infection control performance, confirming the utility of the ecological systems framework. Among the predictors, communication emerged as the strongest factor, followed by safety control and the presence of 3–4 negative‐pressure isolation rooms with anterooms. It should be noted that the reference category for this variable was hospitals with 1–2 negative‐pressure isolation rooms. Therefore, the significant association observed for hospitals with 3–4 rooms indicates a difference relative to the reference group rather than a linear increase in infection control performance as the number of rooms increases. These results emphasize the importance of both interpersonal and structural factors in enhancing infection control performance. Therefore, when designing nursing interventions for ED nurses, it is essential to incorporate strategies that foster effective communication, reinforce individual safety control, and ensure adequate environmental infrastructure. Practical communication enhancement strategies may include regular interdisciplinary briefings and standardized information‐sharing protocols during EID situations.

In this study, communication, representing the mesosystem, emerged as the most influential predictor of infection control performance. This finding aligns with Yi and Cha [17], although the present study reported higher communication scores. The critical role of communication is further supported by Noble et al. [36], who found that mobile communication systems helped reduce nurse fatigue and optimized clinical workflows. An effective response to EIDs requires institutional systems that actively support communication through training, information‐sharing, and feedback. While existing programs have improved general clinical competencies [37, 38], there remains a need for studies that directly evaluate their impact on infection control performance. Thus, communication enhancement programs tailored to ED settings during EID outbreaks should be developed and systematically evaluated.

Safety control, aligned with the microsystem, was the second strongest predictor. Although its effect was statistically significant, its explanatory power decreased in the final model. This attenuation may be explained by a mismatch in measurement specificity: the tool used for safety control captured general perceptions of safety in EDs, not limited to EID‐specific contexts. In contrast, the communication tool was designed based on EID guidelines, better reflecting the study’s dependent variable—EID‐specific infection control performance. This measurement inconsistency may have resulted in an underestimation of the effect of safety control. Therefore, future research should develop and validate EID‐specific tools for assessing safety control. Strengthening safety control has been shown to reduce infection exposure risk [39] and thus should be a key component of nursing intervention programs designed to enhance infection control performance.

Among the work‐related variables representing the macrosystem, only hospitals with 3–4 negative‐pressure isolation rooms with anterooms showed significantly higher infection control performance compared with the reference group (1–2 rooms). In contrast, hospitals with ≥ 5 rooms did not differ significantly from the reference group. Therefore, the findings should not be interpreted as indicating a simple dose–response relationship between the number of isolation rooms and infection control performance. The discrepancy between the post hoc comparison results and the multivariable regression findings may be attributable to the influence of other covariates included in the adjusted model, differences in organizational characteristics across hospitals, or unequal group distributions. Therefore, caution is warranted when interpreting this result. Although other factors—such as dedicated personnel, PPE availability, prior EID experience, and PPE training—were not significant in the final model, they exhibited meaningful associations in preliminary analyses. These effects may have been suppressed by stronger predictors such as communication and safety control. Nonetheless, these work‐related elements are essential for establishing a safe work environment and should be reflected in institutional policy and structural improvements. Higher infection control performance was observed in nurses who worked less than 8 h per day, suggesting that shift length and staffing models are also relevant. Previous studies [17, 23, 40, 41] support the role of negative‐pressure rooms in improving PPE use and infection control. However, since this study was conducted after the acute COVID‐19 phase, further research is needed to determine the optimal number of isolation rooms and appropriate staffing ratios during various stages of EID outbreaks.

Perceived patient safety culture, conceptualized within the exosystem, did not demonstrate a statistically significant association with infection control performance in the final model. Among all predictors, it had the smallest effect size. This result may be attributable to the measurement tool, which was not specifically developed for EID contexts, potentially limiting its sensitivity. Nonetheless, univariate regression showed that patient safety culture perception (*β* = 0.68, *p* < 0.001, adj *R* ^2^ = 0.46) was significantly associated with infection control performance. In addition, the nonsignificant finding for perceived patient safety culture in the final model may indicate that its additional explanatory contribution was limited after accounting for safety control and communication. This suggests that the influence of perceived patient safety culture may overlap with these more proximal predictors, which demonstrated stronger independent associations with infection control performance in the final model.

According to Bronfenbrenner [12], the exosystem refers to social environments that influence individuals indirectly. In this study, safety culture was measured through nurses’ perceptions, which served as an indirect indicator of the unit’s overall culture. This reinforces the need to develop EID‐specific safety culture instruments that can more accurately assess its impact. Supporting literature further validates its importance: Seong [42] reported a positive link between safety culture and infection control performance, and Lee [22] found that safety culture perception reduced infection control fatigue and promoted preventive behaviors. These findings collectively suggest that enhancing nurses’ perception of patient safety culture remains relevant to infection control practices, even if not detected as a strong predictor in multivariate models.

This study has several limitations. First, although participants were recruited from tertiary hospitals across the country, the sample distribution may have been uneven and potentially biased, which limits the generalizability of the findings to all ED nurses in South Korea. Second, the study did not collect information on the actual number of personnel assigned to negative‐pressure isolation rooms at each hospital during the time of data collection. As a result, it was not possible to determine the optimal staffing ratio or use this variable as a basis for structural recommendations. Third, Bronfenbrenner’s ecological systems theory—which guided the study—conceptualizes human development as shaped not only by environmental factors but also by maturation and evolutionary processes over time [12]. However, these aspects were not incorporated into the current study’s design or analysis. Specifically, the study was conducted based on four levels of Bronfenbrenner’s ecological systems framework (micro‐, meso‐, exo‐, and macrosystems) and did not account for the chronosystem, which may have limited the comprehensiveness of the analysis. Fourth, as the study was based on self‐reported questionnaires, the possibility of response bias, including social desirability bias, cannot be excluded. Fifth, several variables, including working hours, the number of EID‐related education, and education for donning and doffing of PPE, were categorized for analysis, which may have limited the ability to capture more detailed relationships among variables.

## 5. Implications for Nursing Management

This study provides practical insights for nurse managers seeking to improve infection control performance among ED nurses during EID outbreaks. The findings indicate that infection control performance is shaped by factors operating at the individual, interpersonal, and organizational levels, and that interventions should be designed accordingly. First, safety control at the microsystem level should be strengthened through regular, scenario‐based training on PPE donning and doffing and unit‐specific safety protocols, regardless of the availability of EID‐specific assessment tools. Second, communication at the mesosystem level identified as the strongest predictor of infection control performance should be enhanced through standardized handover protocols for suspected or confirmed EID cases and regular interdisciplinary briefings. Third, given the nonlinear association observed between the number of negative‐pressure isolation rooms and infection control performance, hospital administrators and nurse managers should ensure that isolation room capacity is matched with adequate staffing ratios and shift scheduling, particularly during periods of surge demand. Fourth, although perceived patient safety culture did not remain significant in the final model, unit‐level efforts to strengthen safety culture through leadership engagement and structured staff feedback may still indirectly support infection control performance. Implementing coordinated, multilevel interventions that integrate individual competency, communication, and organizational infrastructure is essential for strengthening ED nurses’ preparedness for future EID outbreaks.

## 6. Conclusions

In this study, we examined the relevance of safety control, communication, and perceived patient safety culture to infection control performance among ED nurses. The findings showed that infection control performance was positively associated with communication and safety control. Additionally, nurses working in hospitals with 3–4 negative‐pressure isolation rooms demonstrated higher infection control performance compared to those in hospitals with only 1–2 such rooms. These findings suggest the importance of considering individual, organizational, and environmental factors when developing strategies to support infection control performance. Future research should evaluate the effectiveness of interventions targeting these factors in improving infection control performance.

## Author Contributions

**YJ Park:** conceptualization, methodology, data curation, investigation, writing–original draft, and writing–review and editing. **HY Kim**: conceptualization, methodology, data curation, formal analysis, project administration, supervision, writing–original draft, and writing–review and editing. All authors contributed to the preparation of the manuscript.

## Funding

This research received no specific grant from any funding agency in the public, commercial, or not‐for‐profit sectors. Funding for this research was covered by the authors of the article.

## Disclosure

All authors approved the final submitted version.

## Ethics Statement

The study design was approved by the Institutional Review Board of Jeonbuk National University (IRB) prior to data collection (IRB No. JBNU 2023‐08‐012‐005).

## Conflicts of Interest

The authors declare no conflicts of interest.

## Supporting Information

Additional supporting information can be found online in the Supporting Information section.

## Supporting information


**Supporting Information** STROBE Statement.

## Data Availability

The data that support the findings of this study are available from the corresponding author upon reasonable request.
