# Supplementary material for: Infection Control Performance Related to Emerging Infectious Diseases Among Emergency Department Nurses: An Ecological Systems Theory‐Based Cross‐Sectional Study
Source: J Nurs Manag. 2026 Jul 29;2026:6160239. doi: 10.1155/jonm/6160239 (PMC13417014; doi:10.1155/jonm/6160239)
Supplement: Supplementary file 1 — Supporting Information STROBE Statement. [file JONM-2026-6160239-s001.docx]

| STROBE Statement—checklist of items that should be included in reports of observational studies | | | | |  |
| --- | --- | --- | --- | --- | --- |
|  | **Item** |  | **Page** | **Relevant text from** | |
|  | **No.** | **Recommendation** | **No.** | **manuscript** | |
| **Title and abstract** | １ | (*a*) Indicate the study’s design with a commonly used term in the title or the abstract | 1 | A cross-sectional descriptive study was conducted. | |
|  |  | (*b*) Provide in the abstract an informative and balanced summary of what was done and what was　found | 1 | Communication exhibited the strongest association with infection control performance … | |
|  |  | **Introduction** |  |  | |
| Background  /rationale | 2 | Explain the scientific background and rationale for the investigation being reported | 3 | During outbreaks of emerging infectious diseases, maintaining high standards of infection control among ER healthcare workers is particularly challenging due to… | |
| Objectives | 3 | State specific objectives, including any prespecified hypotheses | 5,6 | Introduction: This study aims to comprehensively examine factors influencing ER nurses’ infection control performance regarding EIDs …  2.1 Aims and hypothesis: The study hypotheses were as follows: (H1) | |
|  |  | **Methods** |  |  | |
| Study design | 4 | Present key elements of study design early in the paper | 6,9 | 2.2 Study design: A descriptive, cross-sectional design was adopted.  2.5. Data Collection: Data for this study were collected through an online survey conducted from … | |
| Setting | 5 | Describe the setting, locations, and relevant dates, including periods of recruitment, exposure， follow-up, and data collection | 9 | Data for this study were collected through an online survey conducted from … | |
| Participants | 6 | *Cohort study*—Give the eligibility criteria, and the sources and methods of selection of participants. Describe methods of follow-up  *Case-control study*—Give the eligibility criteria, and the sources and methods of case ascertainment and control selection. Give the rationale for the choice of cases and controls  *Cross-sectional study*—Give the eligibility criteria, and the sources and methods of selection of　participants |  | Convenience sampling was employed to recruit ER nurses working at tertiary hospitals in South Korea. **The inclusion criteria were as follows…** | |
|  |  |  | 6 |  |  |
|  |  |  |  |  |  |
|  |  | (*b*) *Cohort study*—For matched studies, give matching criteria and number of exposed and  unexposed  *Case-control study*—For matched studies, give matching criteria and the number of controls per　case | N/A |  | |
|  | |  |  |  | |
| Variables | 7 | Clearly define all outcomes, exposures, predictors, potential confounders, and effect modifiers．  Give diagnostic criteria, if applicable | 6-8 | The instruments used to measure infection control performance, perceived safety control, communication, perceived patient safety culture, and infection control performance were employed with permission from the original developers. | |
| Data sources／  Measurement | 8* | For each variable of interest, give sources of data and details of methods of assessment (measurement). Describe comparability of assessment methods if there is more than one group | 6-8 | Data were collected using a structured questionnaire composed of five sections. The instruments used to measure infection control performance, perceived safety control, communication, perceived patient safety culture, and infection control performance were employed with permission from the original developers. | |
| Bias | 9 | Describe any efforts to address potential sources of bias |  |  | |
| Study size | 10 | Explain how the study size was arrived at | 6 | The required sample size was calculated using G*Power 3.1.9.7 software. | |
| Quantitative　variables | 11 | Explain how quantitative variables were handled in the analyses. If applicable, describe which  groupings were chosen and why |  |  | |
| Statistical　methods | 12 | (*a*) Describe all statistical methods, including those used to control for confounding | 9,10 | Data were analyzed using SPSS version 28.0... Hierarchical multiple regression analysis was performed... | |
|  |  | (*b*) Describe any methods used to examine subgroups and interactions | 12 | Partial correlation analysis was conducted … | |
|  |  | (*c*) Explain how missing data were addressed | 9 | A total of 227 responses were collected …without any missing data … | |
|  |  | (*d*) *Cohort study*—If applicable, explain how loss to follow-up was addressed  *Case-control study*—If applicable, explain how matching of cases and controls was addressed  *Cross-sectional study*—If applicable, describe analytical methods taking account of sampling　strategy | N/A |  | |
|  |  | (*e*) Describe any sensitivity analyses | N/A |  | |
|  |  | **Results** |  |  | |
| Participants | 13* | (a) Report numbers of individuals at each stage of study—eg numbers potentially eligible, examined for eligibility, confirmed eligible, included in the study, completing follow-up, and analysed | 6 | Convenience sampling was employed to recruit ER nurses working at tertiary hospitals in South Korea. Considering a potential dropout rate of approximately 15%, 243 responses were collected … After excluding 16 participants … data from 227 participants were included in the final analysis. | |
|  |  | (b) Give reasons for non-participation at each stage | 6 | After excluding 16 participants who did not meet the inclusion criteria, data from 227 participants were included in the final analysis. | |
|  |  | (c) Consider use of a flow diagram | N/A |  | |
| Descriptive data | 14* | (a) Give characteristics of study participants (eg demographic, clinical, social) and information on exposures and potential confounders | 10 | The Socio-demographic characteristics and work characteristics of patients are shown in Table 1 and Table 2. | |
|  |  | (b) Indicate number of participants with missing data for each variable of interest | 10-14 | There were no missing data for any variable of interest. | |
|  |  | (c) *Cohort study*—Summarise follow-up time (eg, average and total amount) | N/A |  | |
| Outcome data | 15* | *Cohort study*—Report numbers of outcome events or summary measures over time | N/A |  | |
|  |  | *Case-control study—*Report numbers in each exposure category, or summary measures of exposure | N/A |  | |
|  |  | *Cross-sectional study—*Report numbers of outcome events or summary measure | 11-12 | Table 3 shows the scores of safety control, communication… | |
| Main results | 16 | (*a*) Give unadjusted estimates and, if applicable, confounder-adjusted estimates and their precision (eg, 95% confidence interval). Make clear which confounders were adjusted for and why they were included | 12-14 | Table 4 presents the results of the hierarchical regression analysis. | |
|  |  | (*b*) Report category boundaries when continuous variables were categorized | N/A |  | |
|  |  | (*c*) If relevant, consider translating estimates of relative risk into absolute risk for a meaningful time period | N/A |  | |
| Other analyses | 17 | Report other analyses done—eg analyses of subgroups and interactions, and sensitivity analyses | N/A |  | |
| **Discussion** |  |  |  |  | |
| Key results | 18 | Summarise key results with reference to study objectives | 14 | The findings revealed that communication and safety control were significantly associated with infection control performance, and that ERs with 3–4 negative pressure isolation rooms demonstrated higher performance levels. | |
| Limitations | 19 | Discuss limitations of the study, taking into account sources of potential bias or imprecision. Discuss both direction and magnitude of any potential bias | 17,18 | This study has several limitations. First… | |
| Interpretation | 20 | Give a cautious overall interpretation of results considering objectives, limitations, multiplicity of　analyses, results from similar studies, and other relevant evidence | 15-17 | In this study, communication, representing the mesosystem, emerged as the most influential predictor of infection control performance. This finding aligns with Yi and Cha [14]… | |
| Generalisability | 21 | Discuss the generalisability (external validity) of the study results | 14,17 | This study, grounded in Bronfenbrenner’s Ecological Systems Theory (1979), …  …participants were recruited from tertiary hospitals across the country.. | |
| **Other information** | |  |  |  | |
| Funding | 22 | Give the source of funding and the role of the funders for the present study and, if applicable, for the original study on which the present article is based | Title page |  | |
